# Illumina sequencing-based analysis of free-living bacterial community dynamics during an *Akashiwo sanguine* bloom in Xiamen sea, China

**DOI:** 10.1038/srep08476

**Published:** 2015-02-16

**Authors:** Caiyun Yang, Yi Li, Benjamin Zhou, Yanyan Zhou, Wei Zheng, Yun Tian, Joy D. Van Nostrand, Liyou Wu, Zhili He, Jizhong Zhou, Tianling Zheng

**Affiliations:** 1 State Key Laboratory for Marine Environmental Science, and Key Laboratory of the Ministry of Education for Coastal and Wetland Ecosystems, School of Life Sciences, Xiamen University, Xiamen 361005, China; 2 Department of Computer Science, Stanford University, Stanford, California 94305, USA; 3 Institute for Environmental Genomics and Department of Microbiology and Plant Biology, University of Oklahoma, Norman, OK 730722, USA; 4 Earth Sciences Division, Lawrence Berkeley National Laboratory, Berkeley, CA 94720, USA; 5 School of Environment, Tsinghua University, Beijing 100084, China

## Abstract

Although phytoplankton are the major source of marine dissolved organic matter (DOM), their blooms are a global problem that can greatly affect marine ecological systems, especially free-living bacteria, which are the primary DOM degraders. In this study, we analyzed free-living bacterial communities from Xiamen sea during an *Akashiwo sanguine* bloom using Illumina MiSeq sequencing of 16S rRNA gene amplicons. The bloom was probably stimulated by low salinity and ended after abatement of eutrophication pollution. A total of 658,446 sequence reads and 11,807 OTUs were obtained in both bloom and control samples with Alpha-proteobacteria and Gamma-proteobacteria being the predominant classes detected. The bloom decreased bacterial diversity, increased species evenness, and significantly changed the bacterial community structure. Bacterial communities within the bloom were more homogeneous than those within the control area. The bacteria stimulated by this bloom included the SAR86 and SAR116 clades and the AEGEAN-169 marine group, but a few were suppressed. In addition, many bacteria known to be associated with phytoplankton were detected only in the bloom samples. This study revealed the great influence of an *A. sanguinea* bloom on free-living bacterial communities, and provided new insights into the relationship between bacteria and *A. sanguinea* in marine ecosystems.

Phytoplankton blooms are a worldwide ecological problem due to eutrophication pollution^1^,^2^,^3^,^4^,^5^,^6^, which can stimulate phytoplankton growth and proliferation. The majority of organic matter in the marine environment, particularly dissolved organic matter (DOM), is produced by phytoplankton^7^,^8^,^9^. Free-living bacteria in these environments hydrolyze most of the DOM^10^, preventing its accumulation during periods of high DOM production such as during phytoplankton blooms^11^. The free-living bacteria account for most of the total bacterial production during a bloom^12^. The interactions between phytoplankton and bacteria are often intertwined, with extracellular products being produced by both in coastal marine environments^9^,^13^. Bacteria can stimulate^14^ or inhibit^15^ phytoplankton growth and can even kill phytoplankton^4^,^6^,^16^,^17^. Therefore, it is necessary to understand how phytoplankton blooms affect marine microbial communities, and determine whether microbial communities are able to control phytoplankton blooms.

Many molecular tools have been utilized to investigate bacterial communities associated with phytoplankton blooms, such as 16S ribosomal DNA (rDNA) clone libraries^18^, polymerase chain reaction-denaturing gradient gel electrophoresis (PCR-DGGE)^11^,^19^, flow cytometry^20^, terminal restriction fragment length polymorphism^18^, fluorescence *in situ* hybridization^21^,^22^, metatranscriptomics^23^ and high-throughput sequencing^24^,^25^. These studies reveal the close relationship between specific phytoplankton blooms and their associated bacterial communities, and which bacterial species may be crucial for the regulation of bloom dynamics and succession. For example, algal-derived organic matter has been shown to control populations of Bacteroidetes, Gamma-proteobacteria and Alpha-proteobacteria^21^; and the *Alteromonas* group can greatly influence the flux of organic matter by proliferation during development of diatom blooms, while Bacteroidetes may contribute to bloom decomposition^26^. These studies greatly expanded our understanding of bloom processes as well as our ability to forecast and prevent blooms. The high-throughput sequencing technologies have developed quickly in recent years, and Illumina MiSeq sequencing has become the most popular since it can generate a multi-million sequence reads of partial 16S rRNA genes to meet the throughput demands of environmental microbial ecology studies and reduce cost^27^.

The dinoflagellate *Akashiwo sanguine* is an alga that causes blooms world-wide. It is eurythermal and euryhaline^28^ and commonly causes spring or summer red tides, especially when the seawater salinity and temperature are relatively low^29^. A few studies of this dinoflagellate have been carried out and have shed light on some bloom dynamics. For example, the raphidophyte *Chattonella antiqua* and *A. sanguinea* inhibit each other's growth^30^; the raphidophyte *Heterosigma akashiwo* inhibits *A. sanguinea* via allelochemicals and direct cell contact to influence bloom formation^31^. Xiuning Du *et al.* studied an *A. sanguinea* bloom along the central Oregon coast and made speculations that the algal seed came from the Washington coast where a massive bloom of *A. sanguinea* had been observed^32^; and the potential impact on the environmental quality of estuarine water has also been investigated^2^. *A. sanguinea* is harmful to birds, fish^33^ and abalone larvae^29^. Since 2008, *A. sanguinea* blooms frequently occur off the coast of Xiamen. Little research has been reported on the bacterial communities associated with these blooms except for our previous research based on PCR-DGGE, which suggested that bacteria may play an important role in the negative regulation of *A. sanguinea*-blooms^2^.

In order to examine the free-living bacterial community during an *A. sanguinea* bloom in more detail 16S rRNA genes of the free-living bacteria were sequenced using the Illumina MiSeq. The aims of this study were to find out which free-living bacterial taxa were dominant during the bloom, how the free-living bacterial diversity and community structure were influenced by this bloom, and whether there were any bacterial taxa that could negatively regulate this bloom. The results showed that the *A. sanguinea* bloom significantly changed the free-living bacterial community structure and stimulated most bacterial taxa; however, the bloom decreased bacterial diversity while increasing species evenness.

## Methods

### Study sites and sample collection

**Figure 1:**
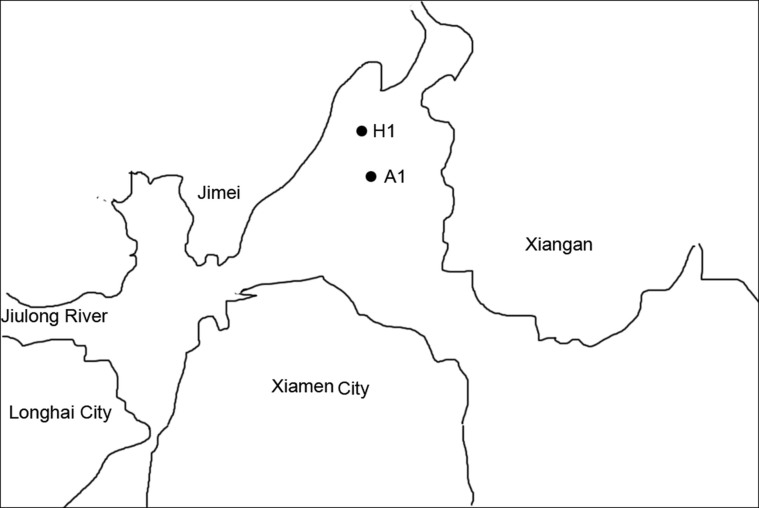
Location of sampling sites. This map was created based on PhotoShop (Version CS5) by CYY.

The A1 bloom site (N 24°35′53.40″, E 118°9'29.67″) and the H1 control site (N 24°36'56.31'', E 118°9'15.92'') were selected in or near the area where an *A. sanguinea* bloom had occurred along the Xiamen coast (Figure 1). Twenty liters of near-surface (0.5 m) sea water was collected from both the bloom and control sites during (31 July, 1–4 August, 2011) and after (7 August, 2011) the bloom. Pre-autoclaved polypropylene sampling vials were used for sampling.

### Environmental parameters

Identification of phytoplankton species, phytoplankton cell counts, concentration of chlorophyll *a*, dissolved inorganic phosphorus (DIP), nitrate, nitrite, and ammonia, the density of *A. sanguinea* and bacteria, chemical oxygen demand (COD) and bacterial productivity were determined as previously described^2^,^34^. Dissolved inorganic nitrogen (DIN) was calculated by summing the molarity of NO_3_^−^, NO_2_^−^ and NH_4_^+^. The N:P ratio was calculated by dividing DIN values by DIP values. The silico-molybdenum blue spectrophotometric method was used for the determination of silicate. The eutrophication index (EI) was calculated as follows: 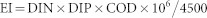
where DIN is the dissolved inorganic nitrogen content in mg/L; DIP is the dissolved inorganic phosphorus content in mg/L; and COD is the chemical oxygen demand in mg/L.

### DNA Extraction and preparation

Water samples (500 mL) were filtered through 5-μm diameter pore-size filters (Millipore, US) to remove particle attached cells. The filtrate was filtered again through 0.22-μm diameter pore-size filters as soon as the samples were taken to the laboratory. Filters were stored at −70°C until analysis (the 5-μm diameter pore-size filters were taken for a comparison study with DGGE). DNA extraction was performed as previously described^2^. DNA quality was assessed using 260/280 nm and 260/230 nm ratios with a NanoDrop ND-1000 Spectrophotometer (NanoDrop Technologies, US). Final DNA concentrations were quantified with PicoGreen^34^ using a FLUOstar Optima microplate reader (BMG Labtech, Germany).

### 16S rRNA gene amplification and Illumina MiSeq sequencing

Primers 515F (5′-GTGCCAGCMGCCGCGG-3′) and 806R (5′-GGACTACHVGGGTWTCTAAT-3′) targeting the V4 hyper variable regions of bacterial 16S rRNA genes were selected^35^. Both forward and reverse primers were tagged with adapter, pad and linker sequences. Each barcode sequence (12 mer) was added to the reverse primer for pooling multiple samples into one run of sequencing. All primers were synthesized by Invitrogen (Life Technologies, USA).

PCR amplification was performed in triplicate using a Gene Amp PCR-System 9700 (Applied Biosystems, USA) in a total volume of 25 μL containing 2.5 μL 10 × PCR bufferII and 0.5 units of AccuPrime™ Taq DNA Polymerase High Fidelity (Life Technologies, USA), 0.4 μM of each primer, and 10 ng template DNA. Thermal cycling conditions were as follows: an initial denaturation at 94°C for 1 min, and 35 cycles at 94°C for 20 s, 53°C for 25 s, and 68°C for 45 s, with a final extension at 68°C for 10 min.

Following amplification, 2 μL of PCR product was used to confirm successful amplification using agarose gel (1%) electrophoresis. The triplicate PCR reactions for each sample preparation were combined and quantified with PicoGreen: 200 ng of PCR product from each sample was pooled for each sequencing run. The pooled mixture was purified with a QIAquick Gel Extraction Kit (QIAGEN Sciences, USA) and analyzed on an Agilent 2100 Bioanalyzer using High Sensitivity DNA Chips (Agilent Technologies, Germany) for size distribution, and then was re-quantified with PicoGreen.

As described in the MiSeq Reagent Kit Preparation Guide (Illumina, USA), the purified mixture was diluted and denatured to obtain an 8 pM sample DNA library, and mixed with an equal volume of 8 pM PhiX (Illumina, San Diego, CA, USA). Finally, 600 μL of the mixture library was loaded with read 1, read 2 and index sequencing primers^35^ on a 300-cycle (2 × 150 paired ends) kit, and run on a MiSeq at the Institute for Environmental Genomics at the University of Oklahoma.

### Assignment of sequence reads to samples

After we obtained the raw sequences, the PhiX sequences were removed. The remaining raw sequences of 16S rRNA gene were sorted and distinguished by unique sample tags. Since each sample had a unique tag, all sequence reads with the same tag were assigned to the same sample. Based on the primer sequences, each sample could be further separated into two (forward and reverse) regions. Finally, the tag, both primers and spacers were trimmed based on Btrim^36^.

### Combination and data preprocessing

Forward and reverse sequences were merged by overlapping paired-end reads using FLASH^37^ with a required overlap length of 10–100 bp. For all 30 samples, the number of merged reads ranged from 16,632 to 36,368 and the average merged read length was 250 bp. Low quality fragments were removed before Chimera detection and removal using U-Chime^38^. For all 30 samples, the number of reads ranged from 13,600 to 31,639. Samples were randomly resampled at 13,600 sequences. OTUs were classified using UCLUST at a 97% similarity level^39^. Taxonomic assignment was performed using the RDP classifier^40^ (http://rdp.cme.msu.edu). All statistical analyses were performed in R^41^. Dissimilarity tests were based on the Bray-Curtis dissimilarity index using analysis of similarities (ANOSIM)^42^, non-parametric multivariate analysis of variance (adonis)^43^, and multi-response permutation procedures (MRPP)^44^. Monte Carlo permutation was used to test statistical significances. Statistics were performed running the Vegan package (v.1.15–1)^45^ in R. The data comparison between the two sites was performed only for samples on 31 July and 2-4August (days 1, 3, 4, and 5 during the bloom) since there was insufficient DNA extracted from the control site on 1 and 7 August.

## Results

### Overview of sequencing analysis

**Figure 2:**
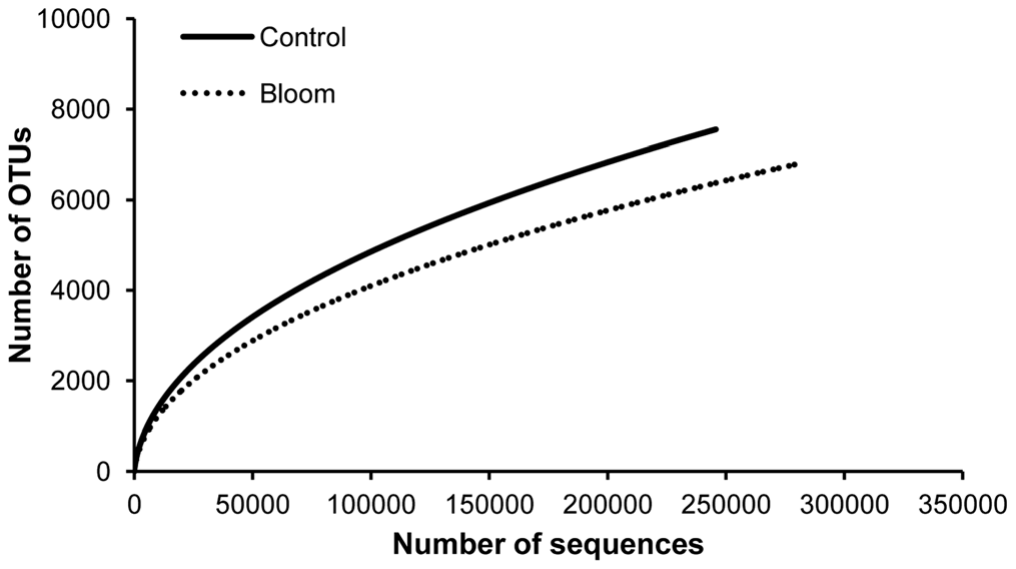
Rarefaction curves of bloom and control samples.

**Figure 3:**
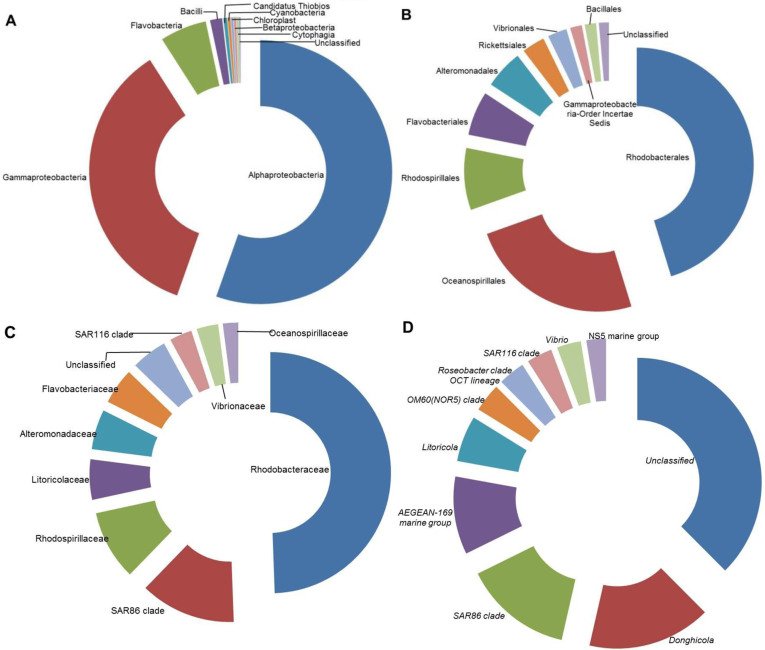
Composition of the top 10 taxa at the (A) class, (B) order, (C) family, and (D) genus level for all samples.

After processing, 658,446 high quality sequences remained with an average length of 253 bases. A total of 11,807 OTUs were generated after clustering at a 97% similarity level and 5,672 OTUs were singletons. Species richness estimates of control and bloom samples were quite high (14,392.85 and 12,882.17, respectively) using the Chao estimator, while the rarefaction curves of control and bloom samples (Figure 2) were still far from saturation. A relatively large number (5,601) of OTUs failed to be assigned into any genus with a confidence level higher than 50% (Table 1), suggesting the presence of many potentially novel bacteria in the sea area near Xiamen. A higher percentage of unclassified sequences were detected in the control area (33.6%) than in the bloom area (23.3%). Archaea accounted for 0.016% (19 OTUs) of the total population, including the phyla Euryarchaeota and Thaumarchaeota. The bacteria were from 30 phyla, 75 classes, 156 orders, 292 families, and 542 genera. Proteobacteria (91%) was the most abundant phylum with 54.8% contributed by Alpha-proteobacteria and 35.1% by Gamma-proteobacteria. The top 10 most abundant sequences at class, order, family and genus levels accounted for 98.9, 92.7, 84.8 and 76.9% of all sequences, respectively (Figure 3).

### Environmental parameters

A prolonged bloom dominated by *A. sanguinea* (mixed with a small amount of *Skeletonema costatum* and *Alexandrium tamarense*) occurred in site A1 from 31 July to 4 August 2011 in the sea area near Xiamen. A distinctly colored border line in the water caused by this bloom appeared between A1 and the control area (H1). The water properties and chemical analysis results are shown in Table 2. Based on the dynamics of algal density, 31 July, 2 and 3 August were during the bloom (B); 1 and 4 August were the bloom peak (BP; named BP1 and BP2, respectively); and 5 August was after the bloom (AB).

All environmental variables showed distinct differences between the bloom and control areas during the sampling periods. Compared to the control area, pH was much higher in A1 although the pH for both A1 and H1 decreased during the bloom. Dissolved oxygen (DO) showed the same pattern except that it was higher in H1 on day 1 and after the bloom. The concentration of suspended particles reached a peak on BP1.

Nitrite and nitrate dynamics were complex but both declined to their lowest levels on BP2 and then recovered by AB. Ammonia nitrogen showed a generally increasing trend in the bloom area except on BP2. DIP concentration was higher in A1, declined to its lowest level on BP2, and then recovered after the bloom. In general, the bloom area had much higher inorganic N and P concentrations and a lower N/P ratio. The silicate concentration was higher in A1 except on BP2 and AB. Silicate concentrations were negatively correlated with *A. sanguine* density, but recovered dramatically by AB. The much higher COD observed in A1, indicating the bloom outbreak, was associated with the level of water pollution.

In general, A1 had a higher chlorophyll *a* concentration than H1 during the bloom because of the high algal density. The EI, based on the inorganic nutrient levels, was higher in A1 than in H1 during the bloom (except for BP2) and indicated that the bloom area had a serious eutrophication status (EI > 1). The EI dynamics were in contrast to the algal density dynamics in that EI decreased as the bloom increased. On day 2 the algal density peaked, decreased and then reached a second peak on day 5. During this same time period, the EI declined to its lowest levels. The EI recovered after the bloom, indicating that this bloom was able to reduce the eutrophication pollution of the sea water. Bacterial density was also higher in the bloom area and total algal density coincided well with *A. sanguinea* cell numbers.

### Overall effects of the *A. sanguine* bloom on free-living bacterial communities

Comparison of the microbial community composition was made between the bloom and control samples for days 1, 3, 4 and 5, these being the only days when the DNA was sufficient in the control area. The 16S rRNA sequences obtained were subjected to random re-sampling at 13,600 sequences for each sample, and sequences appearing in only one of three replicates were removed to minimize errors.

### Bacterial species richness and diversity

**Figure 4:**
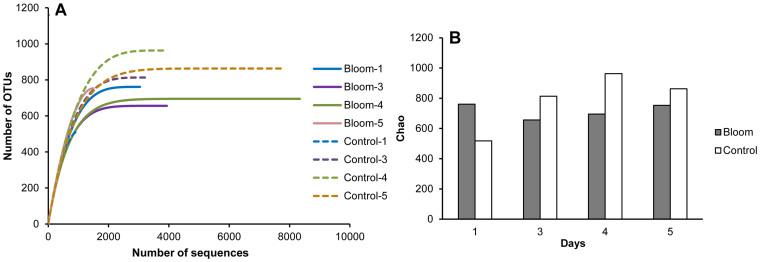
Rarefaction curves (A) and species richness (B) estimates for bloom and control samples using the Chao estimator method.

Rarefaction curves based on a 97% cluster similarity showed a remarkable difference in the free-living bacterial diversity between A1 and H1. The bloom samples showed a lower bacterial diversity than the control samples, except on day 1 (Figure 4); otherwise, the estimated bacterial diversity was more stable during the bloom. However, Shannon diversity showed no significant difference between A1 and H1.

### Bacterial composition and community structure

**Figure 5:**
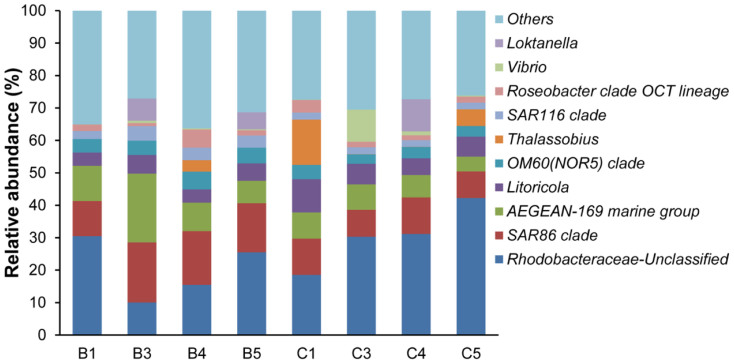
Bacterial community structure at the genus level. Only the top 10 genera are listed, (B): bloom, (C): control.

**Figure 6:**
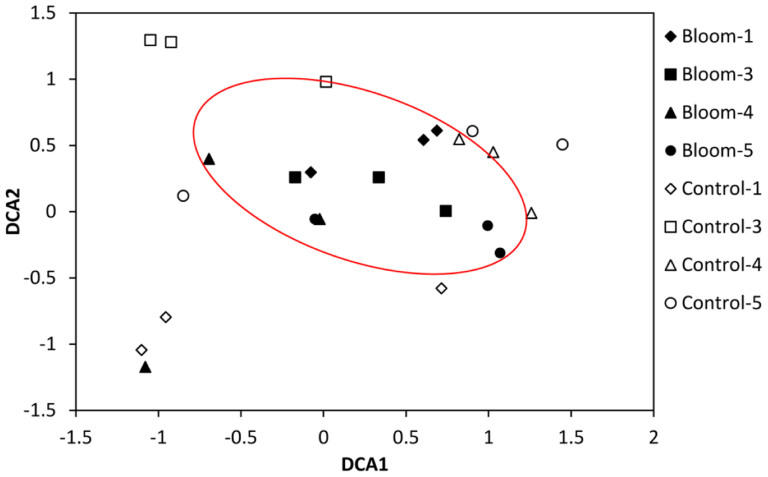
Detrended correspondence analysis (DCA) of the bacterial community.

**Figure 7:**
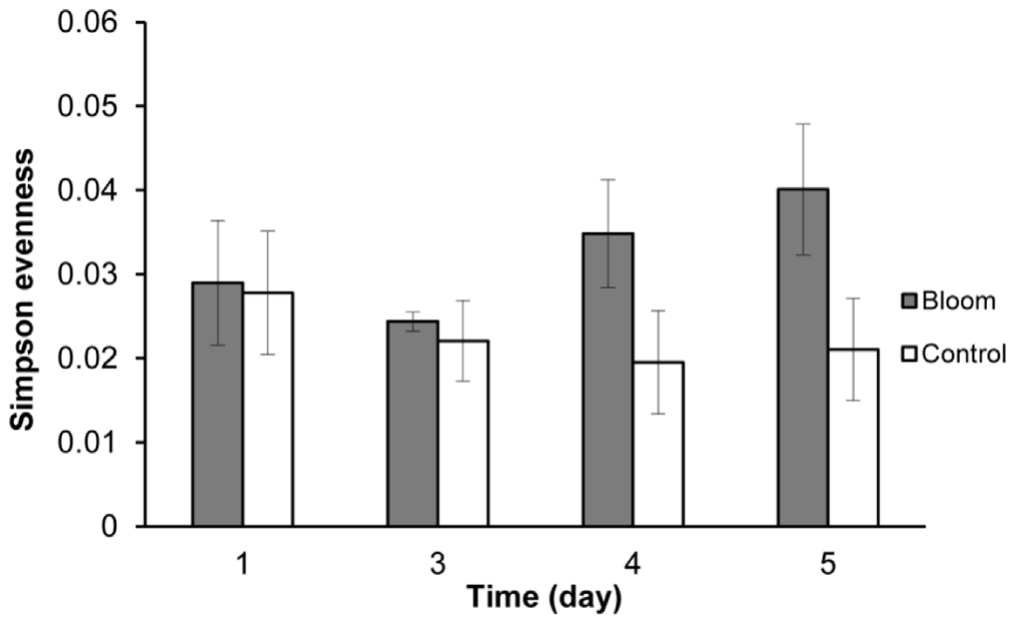
Free-living bacterial Simpson evenness of the bloom and control groups.

The bacterial community structure of the bloom and control samples at the genus level is shown in Figure 5. A dissimilarity test (Table 3) based on the adonis function showed that the free-living bacterial community structure in A1 was significantly different from that of H1, indicating that the bacterial community significantly changed during the algal bloom. Detrended correspondence analysis (DCA) results also showed that samples from the bloom area were clustered together in the center of the ordination plot (Figure 6), while control samples were scattered around the bloom samples, indicating that the bacterial community structures were more similar or stable within the bloom. In addition, bacterial community diversity evenness (Figure 7) was much higher in A1 than in H1. Simpson evenness increased alongside the bloom; while it decreased in the control area.

### Unclassified bacteria

**Figure 8:**
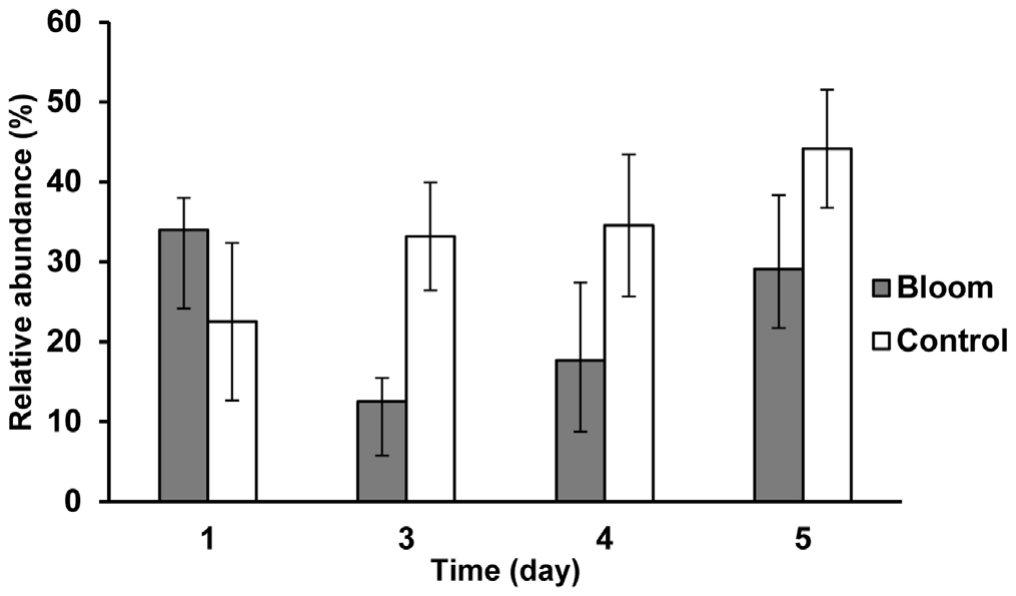
Unclassified sequence ratios of bloom and control areas at the genus level.

Except on day 1, the bloom area had a much lower percentage of unclassified bacteria than the control area, indicating that the bloom environment had more known bacterial species although the percentage increased along with the bloom (Figure 8). At day 3 and later, the unclassified Rhodobacteraceae group and the SAR86 clade were the most dominant groups and, compared to the control area, A1 had fewer Rhodobacteraceae and more SAR86. In addition, A1 had more AEGEAN-169 marine group. In A1, the unclassified Rhodobacteraceae group increased as the bloom increased and decreased again after the bloom. Excluding the top 10 genera, the other genera increased after the bloom.

### Bacteria closely associated with the *A. sanguinea* bloom

T-test results for the top 10 genera showed that, on day 1, A1 had a significantly higher relative abundance of *Loktanella* (P = 0.04) and the NS5 marine group (P = 0.02), but lower *Litoricola* (P = 0.04). On day 3, A1 had more SAR86 (P < 0.01) and SAR116 (P = 0.03) clades, AEGEAN-169 (P = 0.02) and the NS5 marine group (P < 0.01), and OM60 (NOR5) clade (P < 0.01), but fewer *Vibrio* (P = 0.02). On day 4, the bloom area also had fewer *Vibrio* (P = 0.04) and on day 5, A1 had more of the SAR116 clade (P = 0.04).

**Figure 9:**
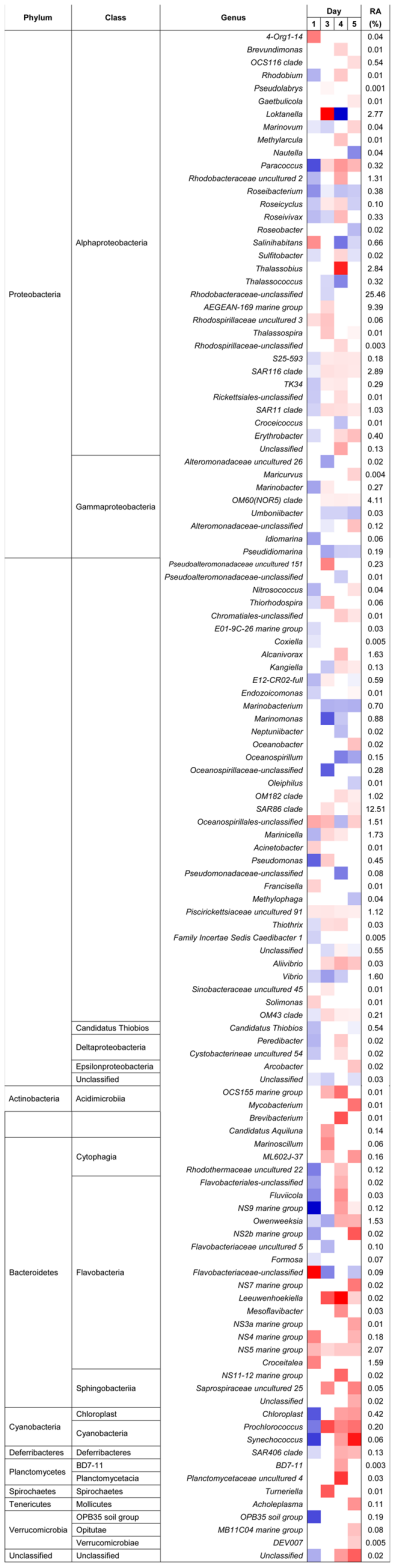
Heat map illustrating the-fold change of genera identified in comparisons between the bloom vs. control areas. Red-orange colors indicate an x-fold increase in abundance in the first comparison indicators, blue colors indicate a decrease. Percentages indicate relative abundances (total = 88.56% of all sequences). The color scale indicates the magnitude of the response ratio.

The response ratio results in Figure 9 showed that a total of 119 genera were significantly different between A1 and H1. The number of taxa that were significantly different between the two sites increased during the bloom. Most of the taxa had much higher relative abundance in A1 except on day 1 (when 12 genera increased and 45 genera decreased). Most known bacteria, such as the predominant genera SAR86, OM60 (NOR5) and the SAR116 clade, and the NS5 marine group, exhibited a higher relative abundance in A1. The Proteobacteria, especially the Alpha-proteobacteria and Gamma-proteobacteria showed the most complex changes.

**Figure 10:**
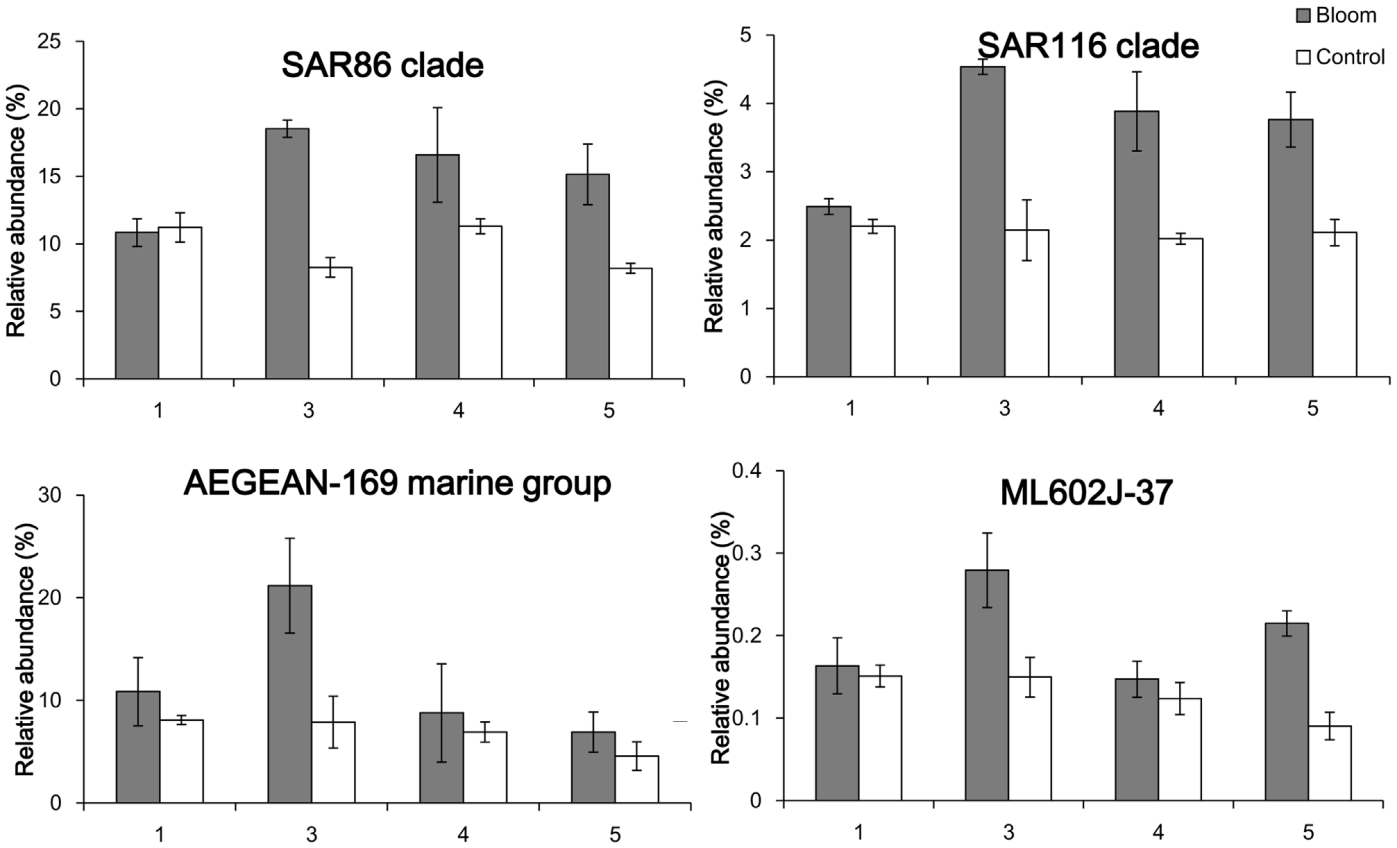
Relative abundance of bacterial genera in the bloom and control areas.

Spearman's correlation analysis between *A. sanguinea* and bacteria showed that many bacteria had significantly positive correlations with this alga, for example the OCS155 marine group (P = 0.02), the *Marinoscillum* (P = 0.03), ML602J-37 of Cytophagia (P = 0.4), the NS3a marine group (P = 0.02), AEGEAN-169 marine group of Rhodospirillaceae (P = 0.04), the SAR116 (P < 0.01) and SAR86 clades (P = 0.048) of the Flavobacteriaceae, *Piscirickettsia* (P = 0.04), and *Acholeplasma* (P = 0.01). In contrast, *Roseibacterium* (P = 0.04), Sulfitobacter (P = 0.048), *Inhella* (P < 0.01) of the *Comamon adaceae,* *Pseudidiomarina* (P = 0.04) and *Bermanella* (P = 0.03) had a negative correlation with *A. sanguinea*. Figure 10 shows the relative abundance dynamics of some related dominant genera during this bloom and they showed a much higher relative abundance in A1. Besides, *Candidatus Aquilunai* strain, the NS3 a marine group, the OM43 clade, *Piscirickettsia* and *Salinihabitans* showed an increase in the bloom. *Salinihabitans* of the Rhodobacteraceae significantly decreased in A1 compared to H1 but it recovered after the bloom, suggesting that an *A. sanguinea* bloom could restrain its growth.

In general, most bacteria, such as the dominant SAR86 and SAR116 clades, and AEGEAN-169 marine group increased during the bloom. These bacteria could be benefitting from the organic matter released from the algae. A total of 37 genera appeared only in the bloom area (Table 4),most from the Proteobacteria.

## Discussion

Algae performing photosynthesis consume CO_2_ and release O_2_. As their numbers increase during a bloom, photosynthesis rates increase resulting in higher pH and DO in the bloom area, which in turn promotes the growth of the phytoplankton^46^. However, as a bloom event ends, algal death and decomposition uses up much of the oxygen in the water and results in decreasing pH and DO. As well as affecting pH and DO, inorganic nutrients were also consumed by the bloom since NO_3_^−^, NO_2_^−^ and DIP concentrations were negatively related to algal density in A1. While these nutrients were consumed by the bloom, the inorganic nutrient concentration was still higher in the bloom than in the control area. The bloom also had a lower N/P ratio indicating that *A. sanguine* required more N than P. Unbalanced consumption of N and P also resulted in the N/P ratio of the water changing within the bloom. Low salinity and high nutrient content from eutrophication pollution are probably important requirements for the *A. sanguinea* bloom formation^28^,^47^. In addition, we found that silicon was important for the growth of *A. sanguine* since the consumption of silicon was concurrent with *A. sanguinea* density during this bloom. Information on silicon consumption by *A. sanguinea* is limited, however, one report notes that silicon concentration decreased alongside an *A. sanguinea* bloom but recovered after the bloom^44^. A bloom might thus relieve eutrophication pollution in sea water by significant consumption of inorganic nutrients. In general, nutrient consumption continued during the bloom. *A. sanguine* consumed a large amount of NO_3_^−^, DIP and SiO_4_^−^ but less NH_4_^+^. While higher COD and EI were needed for bloom formation, the bloom could reduce COD and relieve eutrophication pollution. The bacterial community increased in density in response to the bloom.

The free-living bacterial communities in the bloom and control areas were studied using Illumina sequencing for the first time. Although, in this study, more than 650,000 bacterial sequences and 11,807 OTUs were obtained in the Xiamen sea area, more than half of the OTUs were singletons and the rarefaction curve was unsaturated, indicating the high free bacterial diversity in this coastal area. In addition, 5601 OTUs could not be assigned to any genera with a confidence level higher than 50%, suggesting the presence of abundant novel bacteria in this area. The Proteobacteria was the predominant bacterial phylum while the Alpha-proteobacteria was the predominant class. Alpha-proteobacteria are noted as abundant free-living bacterioplankton both in coastal and open-ocean habitats^48^. Bacteria of both high-nutrient and low-chlorophyll ocean regions and an iron fertilized bloom in the Kerguelen ocean are also dominated by Alpha-proteobacteria^49^. An intriguing finding was the presence of a higher proportion of unclassified sequences in the control versus the bloom area. These unclassified sequences could either have been novel and therefore could not be classified into any of the known lineages or they belonged to less well-studied lineages and so there were few or no sequence representatives. The higher proportion of these unknown sequences in the control area was probably due to the less stable environmental conditions, while the bloom area was more stable. It has been confirmed that certain water stability conditions are necessary for bloom formation and sustainability^50^,^51^,^52^, since hydrology (specifically freshwater discharge, flushing and residence time) greatly influences both nutrient delivery to, and cycling in, affected waters^53^. Additionally, bloom samples would probably be of more interest and would be given greater attention on marine microbial studies. Similarly, in the terrestrial environment, there are more unclassified bacteria in fell-fields (treeless rock strewn areas) compared to vegetated plots in a range of Antarctic habitats^54^.

Compared to the control area, free-living bacterial diversity was lower and evenness much higher in the bloom area, and bacterial composition of the bloom samples was more consistent based on the DCA profile. Since bacterial diversity was relatively stable in the bloom area, this bloom did not change very much in terms of bacterial diversity. In contrast, the bacterial community structure was significantly changed, which is consistent with the results of a study in Norwegian coastal waters where bacterial diversity and community composition were stable during a spring phytoplankton bloom^20^. The bacterial community structure often depends on nutrient composition and phytoplankton exudate composition is species-specific, so that particular phytoplankton results in a specific bacterial community^55^,^56^. The bloom area had a higher proportion of the SAR86 group than the control area, and this group is abundant in bloom communities^18^. SAR86 is a clade of the Gamma-proteobacteria and is one of the most abundant uncultivated microbial groups in ocean surface water^57^,^58^,^59^. SAR86 maybe an aerobic chemoheterotroph based on some complete genomes^58^ and has the ability to consume the wide range of lipids and polysaccharides found in seawater. The biochemical composition of the organic matter produced during a bloom could greatly influence this group, and the results of our study indicated that SAR86 greatly benefitted during *A. sanguine* blooms. The AEGEAN-169 marine group was another dominant group with a higher relative abundance in the bloom area, but there is little relevant ecological information available on this group.

Comparison of the bacterial community structures between bloom and control areas showed that the largest difference occurred on day 3 (during the bloom). However, day 1 (during the bloom) was quite different from the other days. Since phytoplankton can release abundant organic matter that would provide bacteria with carbon and energy sources. The bloom would be expected to enhance most bacteria, particularly dominant species which may be adept at using this resource, such as the SAR116 and OM60 (NOR5) clades, and the AEGEAN-169 and NS5 marine groups. Among these, the SAR116 clade is a unique Alpha-proteobacterial group, and its members are widely found in general marine areas^60^,^61^, as well as in bloom areas^18^,^24^. They could take advantage of the organic matter released from the phytoplankton. The AEGEAN-169 clade is closely related to SAR11, which is extremely abundant in the ocean and feed on dissolved organic carbon and nitrogen. The OM60 (NOR5) clade is a group of Gamma-proteobacteria which is widespread in the euphotic zone of coastal areas^62^,^63^. The abundance of this clade increases in phytoplankton blooms^64^,^65^ and they are positively correlated with chlorophyll fluorescence in some sea areas^66^. The reason for this may be that these bacteria can benefit from organic matter, such as dimethylsulfoniopropionate or dimethylsulfide, released from the phytoplankton^65^.

Among the predominant bacteria which increased in this bloom was the *Candidatus Aquiluna* strain from the Microbacteriaceae, which was first isolated from a lake^67^ while the first strain of this genus to be sequenced was isolated from an Arctic fjord^68^. However, information concerning its relationship to phytoplankton blooms is limited^67^. The OM43 clade of the Methylophilaceae is widespread in the coastal environment^69^,^70^ and is associated with phytoplankton populations and primary productivity^23^. This clade was found during a diatom bloom off the Oregon coast^22^. They may also be able to use the methanol and other C1 compounds produced by phytoplankton^70^,^71^. Information on ML602J-37 of Cytophagia, *Salinihabitans*, *Piscirickettsia* and the NS3a marine group is limited. *Roseobacter* had a higher abundance in the bloom area on day 3 (5.6 ± 0.49%), when algal density was lowest, but this is not as high as has been observed in other studies^11^,^18^,^72^,^73^. Most of the unique bacterial genera that appeared only in the bloom area are known to be associated with algal blooms: *Winogradskyella* is heterotrophic and many isolates are associated with algae^74^,^75^ and phytoplankton blooms^76^; *Aureispira* was was found during a *Microcystis* bloom^77^; SAR202 clade organisms are ubiquitous and they usually occur in mesopelagic and deeper zones^78^,^79^, their appearance in the surface water of bloom areas indicate their presence is closely associated with DOM levels^78^; *Rheinheimera* shows anti-algal activity^80^; *Roseovarius*^81^ and *Bacillus*^82^ are also shown to have algicidal activity; Rickettsia^83^, *Hoeflea*^84^ and *Methylobacterium*^85^ can be symbiotic in algae; *Dinoroseobacter* may have a beneficial relationship with algae^86^ and can be a symbiont of dinoflagellates^87^; and *Colwellia* plays a key role in remineralizing organic matter generated from primary production^88^.

This study found a high bacterial diversity and abundant novel species in the sea area near Xiamen with Alpha-proteobacteria and Gamma-proteobacteria being the predominant classes detected. The low salinity and high eutrophication levels in this area were ideal for the formation of the *A. sanguinea* bloom. This bloom stimulated the growth of those bacterial taxa which could take advantage of the organic matter released by the phytoplankton, such as the SAR86 clade, the AEGEAN-169 marine group and some novel bacterial communities from the Rhodobacteraceae. As the *A. sanguinea* bloom dramatically influenced free-living bacterial communities in many aspects and this study furthered our understanding of its ecological process. Once bacterial communities have changed, so as their ecological functions and which would be studied with functional gene arrays (e.g. GeoChip) in the future.

## Author Contributions

Decision to publish: T.L.Z. and J.Z.Z. Conceived and designed the experiments: C.Y.Y., Y.L., Y.Y.Z., W.Z., Y.T., J.Z.Z. and T.L.Z. Performed the experiments: C.Y.Y., Y.L., B.J.Z., J.D.N., L.Y.W. and Z.L.H. Analyzed the data: C.Y.Y., Y.L., J.D.N., L.Y.W. and Z.L.H. (C.Y.Y. and Y.L., prepared figures 1–6 and Table 1–4. J.D.N., L.Y.W. and Z.L.H. prepared figures 7–10.) Contributed reagents/materials/analysis tools: Y.Y.Z., W.Z., Y.T. and T.L.Z. Wrote the paper: C.Y.Y. and Y.L. All authors reviewed the manuscript.

## Figures and Tables

**Table 1:** Ratio of unclassified sequences at different taxonomic levels

	Relative abundance (%)		
Taxonomy	Total	Bloom	Control
Phylum	0.03	0.04	0.02
Class	0.19	0.17	0.22
Order	1.27	1.08	1.48
Family	4.07	3.89	4.43
Genus	28.87	26.30	31.91

**Table 2:** Properties of samples during the *Akashiwo sanguinea* bloom in 2011 (Only the samples for comparison (bloom vs. control) are shown)

Samples	T	Sal	pH	SP	DO	NO_2_^−^	NO_3_^−^	NH_4_^+^	DIN	DIP	N/P	SiO_4_^−^	COD	CHLa	EI	BACT	*A. sa*	TALG
A1-1	31	24	7.99	2.8	5.25	0.032	0.186	0.009	0.227	0.021	11	0.87	4.66	67.91	4.84	4.60 × 10^11^	1.08 × 10^5^	1.32 × 10^5^
A1-2	31	26	7.97	19.0	4.66	0.029	0.194	0.009	0.232	0.032	7.33	0.84	2.04	51.9	3.32	4.11 × 10^11^	2.05 × 10^5^	2.36 × 10^5^
A1-3	30.8	28	7.89	15.5	4.57	0.030	0.355	- -	- -	0.026	- -	0.95	2.95	23.32	- -	9.96 × 10^11^	1.55 × 10^5^	1.84 × 10^5^
A1-4	30	29	7.87	14.2	4.46	0.030	0.296	0.028	0.354	0.020	18.03	0.90	2.01	33.09	3.09	3.11 × 10^11^	0.81 × 10^5^	0.99 × 10^5^
A1-5	30.5	25	7.84	13.8	3.9	0.013	0.117	0.018	0.147	0.010	15.38	0.57	1.89	52.97	0.59	2.86 × 10^11^	1.70 × 10^5^	1.89 × 10^5^
A1-8	31	30	7.82	10.1	3.51	0.026	0.255	0.078	0.359	0.027	13.20	1.02	1.38	21.3	3.00	- -	0.10 × 10^5^	0.37 × 10^5^
H1-1	31	24	7.89	13.6	6.17	0.043	0.183	0.002	0.228	0.012	18.96	0.79	1.45	38.65	0.88	1.77 × 10^11^	0.18 × 10^5^	0.41 × 10^5^
H1-2	31	24	7.87	10.6	- -	0.027	0.213	0.026	0.266	0.013	20.87	0.56	0.97	28.13	0.74	1.00 × 10^11^	0.08 × 10^5^	0.18 × 10^5^
H1-3	31	25	7.84	10.4	3.65	0.023	0.186	0.031	0.2393	0.024	10.06	0.68	1.26	31.31	1.59	0.75 × 10^11^	0.22 × 10^5^	0.35 × 10^5^
H1-4	30.5	29	7.78	14.6	3.76	0.015	0.194	0.030	0.2385	0.012	20.24	0.79	1.36	21.58	0.85	1.23 × 10^11^	0.63 × 10^5^	0.76 × 10^5^
H1-5	30.5	29	7.75	15.0	3.73	0.023	0.230	0.040	0.2927	0.009	34.02	0.94	1.04	23.14	0.58	2.38 × 10^11^	0.08 × 10^5^	0.14 × 10^5^
H1-8	31.8	26	7.61	13.0	6.13	0.039	0.375	0.122	0.535	0.050	10.61	1.51	1.14	20.81	6.87	- -	0.33 × 10^5^	0.36 × 10^5^

The sample numbers indicate time (days), 1: 31 July; 2–5: 1–3 August; 8: 7 August. T: temperature, °C; Sal: salinity,%; SP: suspended particles, mg/L; DO: dissolved oxygen, mg/L; NO_2_^−^: nitrite nitrogen, mg/L; NO_3_^−^: nitrate nitrogen, mg/L; NH_4_^+^: ammonia nitrogen, mg/L; DIN: dissolved inorganic nitrogen, mg/L; DIP: dissolved inorganic phosphorus, mg/L; N/P: ratio of dissolved inorganic nitrogen and DIP; SiO_4_^−^: silicate mg/L; COD: chemical oxygen demand, mg/L; CHLa: chlorophyll *a*, ug/L; EI: Eutrophication index; BACT: bacterial density, cells/mL; *A. sa*: *Akashiwo sanguinea* density; TALG: total algal density; - -: not available.

**Table 3:** Dissimilarity tests of bacterial communities between bloom and control areas

	Euclidean		Manhattan		Jaccard		Bray	
Time	F	p	F	p	F	p	F	p
Day 1	0.28	<0.01	0.46	0.03	8.67	<0.01	2.95	<0.01
Day 3	0.10	0.07	0.59	<0.01	6.95	<0.01	5.63	<0.01
Day 4	0.22	0.01	0.41	<0.01	8.17	<0.01	2.76	<0.01
Day 5	0.21	<0.01	0.31	0.05	5.81	<0.01	1.81	0.02

**Table 4:** Unique free-living bacteria in the bloom area

Phylum	Class	Genus
Actinobacteria	Actinobacteria	Microbacteriaceae*-DS001*
		*ML602J-51*
Bacteroidetes	Cytophagia	Cyclobacteriaceae *uncultured 128*
	Flavobacteria	*Winogradskyella*
	Sphingobacteria	*Aureispira*
Chloroflexi	SAR202 clade	*SAR202 clade*
Deferribacteres	Deferribacteres	*PAUC34f*
Firmicutes	Bacilli	*Bacillus*
Verrucomicrobia	Opitutae	*Pelagicoccus*
WCHB1-60	WCHB1-60	*WCHB1-60*
Proteobacteria	Alpha-proteobacteria	*4-Org1-14*
		*E6aD10*
		*Methylobacterium*
		*Hoeflea*
		*Dinoroseobacter*
		*Phaeobacter*
		*Roseovarius*
		*OM75 clade*
		*Rhodovibrio*
		*Thalassobaculum*
		*Rickettsia*
		*Caedibacter*
	Delta-proteobacteria	*GR-WP33-58*
	Epsilon-proteobacteria	*Sulfurospirillum*
	Gamma-proteobacteria	*BD2-7*
		*Haliea*
		*Colwellia*
		*Psychrosphaera*
		*Rheinheimera*
		*Trabulsiella*
		*Chromohalobacter*
		*Cobetia*
		*ZD0405*
		*Enhydrobacter*
		*EV818SWSAP88*
		*Piscirickettsia*
		*Solimonas*
